# Parent–Child Vaccination Concordance and Its Relationship to Child Age, Parent Age and Education, and Perceived Social Norms

**DOI:** 10.3390/vaccines11071210

**Published:** 2023-07-06

**Authors:** Pikuei Tu, Danielle Smith, Taylor Parker, Kartik Pejavara, J. Lloyd Michener, Cheryl Lin

**Affiliations:** 1 Policy and Organizational Management Program, Duke University, Durham, NC 27708, USA; 2 Department of Family Medicine & Community Health, Duke University, Durham, NC 27708, USA

**Keywords:** decision-making, vaccine hesitancy, health behavior, COVID-19, children’s health, adolescent, peer influence, social behavior, conformity, attitudes, pediatrics

## Abstract

Researchers established that parental vaccination status often predicts that of their children, but a limited number of studies have examined factors influencing dyadic concordance or discordance (i.e., same or different vaccination status or intent for both members). We investigated how child versus parent age as well as parents’ perceptions of their respective friends’ immunization behavior impacted un/vaccinated parents’ decisions regarding vaccinating their child. An online survey obtained the COVID-19 vaccination status and views of 762 parents of 5–17-year-old children. More than three-quarters of all dyads were concordant; 24.1% of vaccinated parents would not vaccinate their child, with greater hesitancy for younger children and among younger or less educated parents. Children of vaccinated parents and of parents who thought most of their child’s friends were vaccinated were 4.7 and 1.9 times, respectively, more likely to be vaccinated; unvaccinated parents were 3.2 times more likely to accept the vaccine for their child if they believed most of their friends would vaccinate their children. Further, parents who reported that most of their friends were vaccinated were 1.9 times more likely to have obtained the vaccine themselves, illustrating the influence of social norms. Regardless of their own vaccination status, parents of unvaccinated children were more likely to be politically conservative. If communities or circles of friends could achieve or convey a vaccinated norm, this might persuade undecided or reluctant parents to vaccinate their children. Future research should examine the effects of community behavior and messages highlighting social norms on pediatric vaccine uptake.

## 1. Introduction

Child vaccination is critical not only to avoid hospitalization [1,2,3], but also to eradicate vaccine-preventable diseases [4]. Despite its importance, child vaccination rates remain low, with only 55.1% of eligible children in the U.S. vaccinated for the flu [5] and 45% for COVID-19 [6]. The role of parental vaccination status in determining that of their child has been well established, but there have been only a few studies on concordance to date [7,8]. Concordance (or discordance, the opposite) in this research refers to when a parent and child have the same (or dissimilar) vaccination status or intention. Considering the HPV vaccine, parents generally have a higher preference for their children’s vaccination compared to children themselves [9], and parent and child accounts of vaccine decision-making disagreed 57% of the time [7]. One study indicated a greater likelihood of HPV and influenza vaccination amongst children of vaccinated parents [8]; another reported that some older teens decided to get vaccinated for COVID-19 independent of their parents [10]. However, little is known about the factors contributing to these vaccination concordance or discordance dynamics.

The decision to obtain a vaccination is complex, with individual as well as external motivators and barriers. Vaccine hesitancy, defined as the “delay in acceptance or refusal of vaccination despite availability of vaccination services [11]”, is a common phenomenon for both adults and children for many diseases, including HPV [12], seasonal influenza [13], and, most recently, COVID-19 [14]. The literature indicates a myriad of potential influences on vaccine behavior, including demographics such as age and race [10,14,15,16], peers’ vaccine behavior or social expectations [16,17,18,19], and political ideology [16,20,21,22]. Younger parent age and lower level of education were found to be associated with higher levels of vaccine hesitancy [23,24,25,26]. Notably, parents and children generally express similar reasons for hesitancy, centering on low perceived disease risk [12], low perceived need for or value of the vaccine [12,15], and fears of side effects or long-term adverse reactions [27,28,29,30,31]; a child’s young age is often an additional source of reluctance for parents [7,12]. More extrinsically, social norms affect attitudes and receptivity [32], including parents’ decisions to vaccinate their child [18]. For example, perceiving support for the HPV vaccine from parents, friends, and doctors influenced college students’ intention to obtain it [17]. Concerning the COVID-19 vaccine, studies indicated an association between norms and intention to obtain the vaccine in youths and adults [19,33], yet another reported that providing young adults information about pro-vaccine social norms did not motivate vaccine uptake more than information provided by health authorities [34]. Further, a correlation appears to exist between individuals’ political ideology and their trust in science across studies of different vaccines, as conservatives seem less likely to believe in preventative measures and less likely to hold pro-vaccination beliefs than liberals [20,21,22,35,36]. Since parents often make vaccine decisions for their young children and may similarly influence their adolescents’ vaccination choice, these factors could further impact the likelihood of parents vaccinating their children and correspondingly dyadic vaccination decision concordance.

Not all vaccinated parents vaccinate their children, and not all unvaccinated parents are unwilling to vaccinate theirs [37,38]. There is a limited understanding regarding factors associated with parents’ hesitancy over vaccinating their child despite being vaccinated themselves, as well as adolescents of unvaccinated parents obtaining the vaccine. Therefore, it is important to examine how these factors vary between parents of different vaccination statuses. In this study, we identified and analyzed factors related to dyadic COVID-19 vaccination concordance and discordance, focusing on the age of the child compared to the age and educational level of the parents, perceived social norms among both the child’s and parent’s peers, and the parents’ political ideology. Though the infection rates have drastically decreased in the past months and as of 11 May 2023, the U.S. no longer categorizes COVID-19 as a Public Health Emergency [39], the threat of the virus persists. According to the most recent report on 29 April 2023, the numbers of reported cases in groups under the age of 18 are still at 9.4 to 18.8 per 100,000 people, accounted for up to 15.2% of all cases in some regions of the country [40]. We aim to build upon the existing literature describing how various factors are associated with and contributing to child vaccination uptake by also considering the vaccination status of the parent to help more effectively encourage acceptance for pediatric vaccines.

## 2. Methods

### 2.1. Participants and Data Collection

We designed an online survey and, through a survey panel company (Centiment, Denver, CO, USA), obtained a sample of parents with children aged 5–17 living in the United States. Centiment recruited its panel from social media sites; eligible participants who completed the survey were offered a small payment as an incentive (or compensation) that they could either receive via Paypal or donate to a nonprofit organization of their choice. The questionnaire assessed demographic information and a range of factors potentially associated with COVID-19 vaccine uptake, including beliefs and questions related to their child’s vaccination. If participants had multiple children, they were asked to consider the youngest child in responding to the questions. The survey was conducted between October 21 and 5 November 2021. We obtained approval for the study from the Duke University Institutional Review Board, and all participants provided informed consent prior to taking the survey.

### 2.2. Measures

The key outcome variable was parent–child dyadic vaccination concordance (further referred to as both vaccinated or both unvaccinated) or discordance (one vaccinated and the other unvaccinated), defined as the parent and child having the same/different vaccination status or intention to get vaccinated. Vaccination status was assessed using the survey questions “Do you plan to get or have you already received the COVID-19 vaccine?” for parents and “Do you plan to have the child receive the COVID-19 vaccine (when it becomes available to the age group) or is the child already vaccinated?” regarding their child. Concordance was determined by whether the parents were vaccinated (or not) and if they intend to or have already vaccinate(d) their child. We used the responses to these two questions to create the following four groups:

- Group 1: vaccinated parent who intends to or has vaccinate(d) their child (Vv).
- Group 2: vaccinated parent who has not vaccinated their child or does not intend to (Vu).
- Group 3: unvaccinated parent who intends to or has vaccinate(d) their child (Uv).
- Group 4: unvaccinated parent who has not vaccinated their child or does not intend to (Uu).

In denoting the four groups of parent–child dyads, the first, upper-case letter “V” or “U” represents the vaccinated or unvaccinated status of the parent; the second, lower-case letter “v” or “u” represents the vaccination status of the child (or parent’s intention to vaccinate the child). We further categorized Groups 1 and 4 as concordant and Groups 2 and 3 as discordant.

Child age was reported by the parents in the ranges of 5–8, 9–11, 12–15, and 16–17 years old. Participant (parent) age was reported in the ranges of 18–22, 23–29, 30–39, 40–49, 50–64, and 65+ years old. Participants self-reported their level of education as “some high school”, “high school diploma or equivalent (e.g., GED)”, “some college or trade school”, “2- or 4-year college degree”, or “graduate/professional degree”.

We assessed perceived social norms related to vaccination using three questions: (1a) “Do you think most of the child’s friends would get the COVID vaccine when it becomes available to their age group?” for parents of children 5–11 years old or (1b) “Did most of the child’s friends get the COVID vaccine already?” for parents of children 12–17 years old; (2) “Do you think most of your friends who have children would vaccinate their children?” (all parents); and (3) “Did most of your friends get the COVID-19 vaccine?” The first two questions were designed to examine the potential influences of the child’s and parent’s peer behavior, respectively, on parental decision for their child as well as concordance; the third question aimed to evaluate the association between friends’ vaccination status and that of the parents themselves. Parallel questions 1a and 1b were intentionally structured in this way because the COVID-19 vaccine was still pending Emergency Use Authorization for children aged 5–11 when the survey was initially launched. All items had the same response options of “Yes”, “No”, “About half”, and “I am not sure”.

We also asked participants to indicate their political orientation and, based on their responses, grouped them into “Republican/Leaning Republican”, “Democrat/Leaning Democrat”, and “Independent/Unaffiliated”. Participants who responded with “Something else/I am not sure” were excluded from analysis when examining the relationship to vaccine behavior.

An expert panel provided consultation for the design of the survey questions, with concepts generated from an extensive literature review and research team discussions. Prior to finalizing the survey, a pilot test was conducted with a diverse group to assess the clarity and validity of the questions and answer choices. Some of the wording and order of answer options were subsequently modified based on the responses from the pilot study.

### 2.3. Data Analysis

We first ran chi-square tests to determine separately the association between variables (e.g., parent and child vaccination status). Risk ratios (RR) were calculated to compare between parents with different vaccination positions and perceptions in accepting the vaccine for their child. Single-sided *t*-tests were run comparing Groups 1 (Vv) and 2 (Vu) to examine whether vaccinated parents not vaccinating their child was related to a particular variable; Groups 3 (Uv) and 4 (Uu) were similarly examined to assess whether a child being vaccinated, despite their parent being unvaccinated, was associated with the variable. Single-sided *t*-tests were used over two-sided *t*-tests to determine significance in the directionality of the difference of the means rather than just significance in the difference of the means [41].

For each of the six independent variables of interest (child age, parent age and education, perceived vaccine behavior of parent’s and child’s friends, and political orientation), additional chi-square tests were conducted for each possible value to compare the frequencies of Groups 1 vs. 2 (vaccinated parents vaccinating their child or not) and Groups 3 vs. 4 (unvaccinated parents vaccinating their child or not); any pairs of frequencies that were not significantly different from one another were noted in the tables. Furthermore, two simple logistic regression models were run to test how well the individual variables predict concordance status and child vaccination status, respectively. Finally, two multiple logistic regression models estimated the relative influence of these variables on concordance status, separating vaccinated and unvaccinated parents. Two additional multiple logistic regression models were run on child vaccination status: the first included all six predictor variables and the second added parent vaccination status as an additional predictor. All statistical data analyses were completed using R Studio (Version 1.4.1103).

## 3. Results

### 3.1. Descriptive Statistics of Concordance/Discordance Groups

We obtained responses from 762 parents with children between the ages of 5 and 17 years old. The study sample consisted of 355 men (46.6%), 402 women (52.8%), and 5 respondents (0.7%) who self-identified as another gender or preferred not to answer. The racial and ethnic breakdown was 529 White (69.4%), 84 Black (11.0%), 72 Hispanic (9.5%), 45 Asian (5.9%), 13 Native American (1.7%), and 19 (2.5%) who identified as another race/ethnicity or preferred not to say. The majority were between 30 and 49 years old (75.7%, n = 577).

The four dyadic combinations varied in size (Table 1). About 21.3% of the surveyed parents were discordant (Groups 2 and 3). There was a significant association between parent and child vaccination status (X^2^ = 243.64, df = 1, *p* < 0.001). Parents who were vaccinated were 4.7 times more likely to vaccinate their child against COVID-19 than unvaccinated parents (75.9% vs. 15.9%, t = −20.06, df = 571.01, *p* < 0.001).

### 3.2. Child Age, Parent Age, and Parent Education

Overall, parents were more likely to vaccinate adolescent children than younger children (64.6% for ages 12 and above vs. 43.7% for ages 5–8; Table 2a). A simple logistic regression indicated that as child age increases by one age-group, the odds of a child being vaccinated increase by a factor of 1.4 (β = 0.32, 95% CI: 1.21–1.58, *p* < 0.001). Vaccinated parents (Groups 1 and 2) with younger children were less likely to vaccinate their child (t = −3.74, df = 196.67, *p* < 0.001). However, the same analysis of unvaccinated parents (Groups 3 and 4) did not indicate a significant relationship (t = −1.12, df = 53.63, *p* = 0.13). Chi-square analysis comparing Groups 1 (Vv) vs. 2 (Vu) and Groups 3 (Uv) vs. 4 (Uu) within each child age group indicated that all frequencies in vertical pairs were significantly different from one another.

Regarding parent age, those of unvaccinated children were, on average, younger than parents of vaccinated children (t = −3.95, df = 703.1, *p* < 0.001). Comparing within the pairs of dis/concordant groups, even among vaccinated parents (Groups 1 and 2), those who were younger were less likely to vaccinate their child (t = −3.10, df = 181.41, *p* = 0.001); however, for unvaccinated parents (Groups 3 and 4), there was no difference in parent age in relation to whether they would vaccinate their child (t = 0.24, df = 49.66, *p* = 0.404; Table 2b). Regression models estimated that as the age of the parent increased by one age-group, the odds of the child being vaccinated increased by a factor of 1.4 (*β* = 0.32, *p* < 0.001, 95% CI: 1.17–1.63).

Across all parent participants, those who did/would not vaccinate their child had less education on average than those who did (t = −6.41, df = 687.63, *p* < 0.001). Simple logistic regression estimated that a single-step increase on the parent education scale led to a 1.5 times increase in the likelihood of the child being vaccinated (*β* = 0.42, *p* < 0.001, 95% CI: 1.33–1.74). A similar trend was observed in vaccinated parents: parents of vaccinated children had a higher education on average than parents of unvaccinated children (t = −1.91, df = 188.31, *p* = 0.029). However, unvaccinated parents showed no significant correlation between their education level and child’s vaccination status (t = −1.4, df = 51.78, *p* = 0.084; Table 2c). Chi-square analysis showed a significant difference comparing the frequencies of Groups 1 (Vv) vs. 2 (Vu) and Groups 3 (Uv) vs. 4 (Uu) within each level of education except one: vaccinated parents with “some high school” showed no statistical difference in the likelihood of vaccinating their child (Χ^2^ = 0.08, df = 1, *p* < 0.782).

### 3.3. Perceived Social Norms

We investigated parent perceptions of vaccination behavior of both the child’s and parent’s peers, respectively, in association with their decision to vaccinate their child (child vaccination status) and concordance. Among vaccinated parents, those who did/would vaccinate their child (Group 1-Vv) were more likely to think that most of their child’s friends would be vaccinated compared to those who did/would not vaccinate their child (Group 2-Vu; 44.1% vs. 22.0%; Χ^2^ = 62.51, df = 3, *p* < 0.001), and the same contrast was observed between parents in Groups 3 (Uv) vs. 4 (Uu) (42.5% vs. 6.6%; Χ^2^ = 40.18, df = 3, *p* < 0.001; Table 3). In addition, chi-square analysis of the vertical pairs showed that among the parents who did not think their child’s friends have been/will be vaccinated, the proportion of people in Groups 1 (Vv) and 2 (Vu) did not differ significantly. Similarly, for those who believed their child’s friends have been/will be vaccinated, the proportion in Groups 3 (Uv) and 4 (Uu) did not differ significantly. Logistic regression analysis confirmed that parent perception of children’s friends’ vaccination statuses predicted parent decision to vaccinate their child (*β* = 1.32, *p* < 0.001, 95% CI: 2.97–4.83): a one-unit increase in perceived child’s friends’ vaccination status was associated with a 3.7-times increase in the odds of a child being vaccinated, regardless of parents’ vaccination status.

Furthermore, relating to parents’ own peers, among unvaccinated parents, compared to those who did/would not vaccinate their child (Group 4, Uu), those who vaccinated or planned to vaccinate their child (Group 3, Uv) were more likely to report that most of their friends would also vaccinate their children (6.2% vs. 50.0%; Χ^2^ = 59.90, df = 3, *p* < 0.001); we observed a similar contrast between Group 2 (Vu) and Group 1 (Vv) parents (24.4% vs. 55.4%; Χ^2^ = 78.76, df = 3, *p* < 0.001; Table 3). For parents who believed their own friends have vaccinated or will vaccinate their child, the proportion in Groups 3 (Uv) and 4 (Uu) did not differ significantly. Simple logistic regressions further showed that an increase in the perception of friends vaccinating their children increased the odds of the participant vaccinating their child by a factor of 5.3 (*β* = 1.67, *p* < 0.001, 95% CI: 4.11–6.97).

Examining the risk ratios between the group that vaccinated their child and the group that did not, parents who believed most of the child’s friends were (or would get) vaccinated were 1.9 times more likely to have their child vaccinated (75.6% vs. 40.2%). Conversely, parents who thought most of their own friends were not vaccinating their children were 0.5 times as likely to vaccinate their child (35.0% vs. 74.8%). More specifically, unvaccinated parents were 3.0 times more likely to vaccinate or plan to vaccinate their child if they thought most of the child’s friends were getting vaccinated (32.3% vs. 10.6%) and were 3.2 times more likely to do so if they perceived that their own friends would vaccinate their children (31.1% vs. 9.6%, Table 3). Furthermore, parents themselves were 1.9 times more likely to be vaccinated if they believed most of their friends received the COVID-19 vaccine (75.6% vs. 40.1%).

### 3.4. Parent Political Orientation

Political orientation was associated with both parent and child vaccination statuses: vaccinated parents as well as parents of vaccinated children were more likely to be Democrat/Leaning Democrat than those unvaccinated or having unvaccinated children (50.7% vs. 27.9% for parents and 55.7% vs. 27.2% for children). Simple logistic regression showed a significant relationship between parent political orientation and child vaccination status (*β* = −0.61, *p* < 0.001, 95% CI: 0.45–0.65). More specifically, among vaccinated parents, those who would not vaccinate their children (Group 2—Vu) were more frequently than those would (Group 1—Vv) to self-report as Republican/Leaning Republican (t = 3.80, df = 181.91, *p* < 0.001). Conversely, among unvaccinated parents, those who vaccinated their child (Group 3—Uv) were more likely to be Democrat/Leaning Democrat than their counterparts who would not vaccinate their child (Group 4—Uu) (t = 3.49, df = 47.57, *p* < 0.001; Table 4). Group 4 (Uu) had the highest proportion of Independent/Unaffiliated at 31.4%. Chi-square analysis comparing the frequencies of Groups 1 (Vv) vs. 2 (Vu) and the frequencies of Groups 3 (Uv) vs. 4 (Uu) within political orientation categories showed statistical significance for all pairs.

### 3.5. Multiple Logistic Regressions

Two multiple logistic regressions (Models I and II) tested the predictive capability of all six variables on concordance status. The first regression assessed the potential indicators for the vaccinated parents (R^2^ = 0.25, *p* < 0.001); child age, political orientation, and both social norm variables were significant predictors of concordance status, with parent perception of their own friends’ decisions to vaccinate their children having the strongest influence (*β* = 0.97, *p* < 0.001, 95% CI: 1.00–1.81); parent age and education were insignificant. The second regression was for the unvaccinated parents (R^2^ = 0.37, *p* < 0.001); parents’ perception of their friends’ decision to vaccinate their children was the only significant indicator (*β* = −1.48, *p* = 0.002, 95% CI: 1.0–4.7; Table 5).

Two more multiple logistic regressions (Models III and IV) were run on child vaccination status on all responses; one included the six variables of interest and the other added parental vaccination status as an additional indicator. The third regression showed that all variables except parent age were significant predictors (R^2^ = 0.3683, *p* < 0.001). Parent’s perception of friends’ intent to vaccinate their children again had the strongest influence on a participant’s decision whether to vaccinate their child (*β* = 1.25, *p* < 0.001, 95% CI: 2.34–5.39), followed by political orientation (*β* = −0.58, *p* < 0.001, 95% CI: 0.41–0.75; i.e., children of Republican-leaning parents were less likely to be vaccinated) and child’s friends’ vaccination statuses (*β* = 0.55, *p* = 0.009, 95% CI: 1.14–2.62; Table 6). For the fourth regression, the additional predictor improved the model fit (R^2^ = 0.4329, *p* < 0.001), with parent vaccination status showing the strongest influence on the likelihood of a parent vaccinating their child (*β* = 1.95, *p* < 0.001, 95% CI: 3.71–13.61); perception of friends’ intent to vaccinate their children became the second most important indicator (β = 1.14, *p* < 0.001, 95% CI: 2.01–4.97), followed by political orientation (*β* = −0.55, *p* < 0.001, 95% CI: 0.42–0.79). Parent age, parent education, and child friends’ vaccination status became insignificant in predicting child vaccination status (Table 7).

## 4. Discussion

By concurrently examining the vaccination statuses of parents and their children, our study offered a more in-depth look into individual and peer factors associated with parents’ decisions concerning pediatric immunization and presented the differences in variable weights across vaccinated versus unvaccinated parents. The finding that 75.9% of vaccinated parents vaccinated or were planning to vaccinate their children compared to only 16.1% of unvaccinated parents reinforced the significant role of parental vaccination status. The expanded multiple regression also confirmed it as the strongest influence on child vaccination. Our analysis further indicated that the vaccination status of the parent can change the association of child age, parent age, and parent education with the parent’s decision to vaccinate the child and correspondingly the concordance of the dyad.

Previous researchers have noted that a child’s younger age increases parental hesitancy [12,15,42] and that parents of younger ages and lower levels of education are less likely to vaccinate their child [23,24,25,26]. We demonstrated that these correlations are more nuanced than they seem, distinguishing that these relationships existed among vaccinated parents but not those unvaccinated. When testing each of the variables independently, all showed a significant relationship to child vaccination status. However, when testing all six potential indicators in a multiple logistic regression model, parent age had no effect on child vaccination status and both parent age and education were insignificant in predicting concordance; on the other hand, child age remained relevant across different models.

Likewise, political ideology persistently impacts vaccination choices for parents as well as their child, posing greater influence than other demographic variables. Parents who did not vaccinate their child were more likely to be politically conservative, even when the parents were vaccinated themselves. This parallels the national trend of Republican-voting counties reporting significantly lower vaccination rates than Democratic-voting counties in the 2020 Presidential election [22]. To address vaccine hesitancy or refusal within the more conservative population, there should be a greater emphasis on protecting others, as research has indicated that messaging about protecting friends and family was more effective in persuading Republicans to receive vaccination [36].

Social norms are well-established predictors of health behaviors, including vaccination intention [19,43]. Nevertheless, to our knowledge, this is the first study to examine the influence of parents’ perceptions of both the vaccination status of their child’s friends and the attitudes of their own friends on vaccinating their children, specifically in relation to parent–child concordance. In our study, a parent’s perception of their peers’ intentions to vaccinate their children consistently showed a strong impact on the decision to vaccinate one’s own child as well as on concordance across both vaccinated and unvaccinated parents, underscoring the pull of social norms on vaccine behavior. The importance of parent peer’s behavior also outweighed child’s friends’ vaccination status, signaling that one’s immediate social circle takes precedence in determining their decisions. This interestingly further illustrated the unique similarity between unvaccinated and vaccinated parents in deciding to vaccinate their child.

In addition, the results showed that both perceptions often aligned with the vaccination status of their own child, regardless of the parents’ vaccination status. Especially worth noting was that even parents who did not accept the vaccine for themselves were at least three times more likely to vaccinate their child if they perceived a norm of vaccination either among the child’s friends or their own friends. Having more vaccinated friends also nearly doubled the chance of a parent being vaccinated. Conversely, vaccinated parents may be dissuaded and thus delay or refuse vaccinating their child if they observe hesitancy in their circle of friends. One potential explanation is that individuals are likely influenced by or tend to conform to the attitudes or behavior of the individuals around them and undertake similar vaccination actions. This influence was described by the psychology theory of observational learning [44] and supported by previous research involving the HPV vaccine [17] and other immunizations [18,32]. On the other hand, parents may choose to associate with those having similar attitudes or ideology [45,46,47] and, in this case, find validation for their position on vaccination. Considering routine vaccination or even the event of a future pandemic, promotional programs could leverage such social influence to persuade undecided or resistant parents in encouraging pediatric uptake. At the same time, caution should be taken as the clustering of negative attitudes (i.e., opposing vaccines) within friend groups may pose a challenge to interventions. Ultimately, for discordant dyads, unvaccinated and vaccinated parents came to different decisions for themselves and their children due, in part, to the same phenomenon: perceptions of their peers’ behavior and their child’s community. This further demonstrates the power of social norms on health behaviors and the choices parents make for their children. Future studies could help distinguish between the perception of a norm and the realities of attitudes and behaviors in a social group. Examining the effect of community-based messaging on pediatric uptake would be informative. Recognizing its impact in encouraging health behaviors, communities and close social circles conveying vaccination as a norm could convince reluctant parents to vaccinate their children. Health authorities and researchers could also investigate the utilization of peer pressure or social expectation as motivators to adopt or reject a vaccine. Improving this understanding could help raise vaccine acceptance for COVID-19 and other diseases, including in the event of a future pandemic, as social responses and potential opinion clustering influence pediatric vaccine decision-making.

### Limitations and Future Directions

One limitation of our survey was that the parents’ children and their beliefs related to the COVID-19 vaccine were not included for paired analysis. However, responses from parents about their child in addition to their opinions allowed the examination of concordance in this analysis not reported in most previous research on parental vaccination decisions. In addition, we sorted parents by vaccination behavior but included intent along with behavior for children’ status. This was due to the unknown timing of the COVID-19 vaccine approval for younger age groups when the survey was launched on 21 October 2021; the vaccine was approved for children 5–11 years old on 29 October 2021. Parents’ decisions might have changed as more safety data were published and children were vaccinated. Future research should look to compare parents’ decisions and intentions over time. Additionally, respondents who chose to participate were self-selective in nature and may have exhibited social desirability bias in answering certain questions. This could potentially skew some of the results and may have misrepresented or not fully demonstrated parents’ willingness to get their child vaccinated. The polarization of COVID-19 vaccine issues may also have altered participants’ attitudes toward the subject. Furthermore, although anticipated, the number of unvaccinated parents with a vaccinated child was small, which might have restricted our ability to determine statistical significance of Group 3-related associations. Finally, a small incentive was provided to recruit participants to complete the survey, which could have potentially increased respondence among certain demographics [48]. However, as offering incentives is a common approach to improve response rates and a practical method to compensate for participants’ time in research, this limitation is not specific to this study [49].

Our survey participants came from all regions of the United States but were not asked about the type of community they lived in (rural, urban, etc.). Future researchers should examine differences in vaccine access and political climate relating to receptivity across counties or states to compare parental intention and subsequent child vaccination, as the location of residence is another important variable in the complex discussion of vaccination and resources [50,51]. Additionally, initiatives should be taken to establish more formal reporting of parent–child dyads to allow bidirectional analysis of the observed factors to increase our understanding of parental decisions concerning COVID-19 as well as other well-established vaccines.

## 5. Conclusions

Our study’s consideration of parent vaccination position in examining predictors of pediatric uptake adds to the knowledge of dyadic concordance to help inform more customized communication campaigns. Parents are more likely to vaccinate their child if they themselves are vaccinated. The likelihood of not vaccinating a child and that of discordance between COVID-19 vaccinated parents and their children was increased by the young age of the child, low vaccine uptake by the individuals around them, and parent self-identification as politically conservative. To achieve higher pediatric vaccination rates, messaging around immunization should pay additional attention to parents of young children and, at the same time, consider parents’ own vaccination status as well as perceptions of social norms to better tailor the strategies. Such messaging, with potential applications to both routine vaccination and future pandemics, should aim to increase interest in public health by emphasizing the vaccine’s role in protecting family and friends and highlighting not only the benefits but also the prevalence of vaccination.

## Figures and Tables

**Table 1:** Parent–Child COVID-19 Vaccination Concordance Categorization (n = 762) *.

		Child Vaccination Status	
		*Vaccinated or* *getting vaccinated* n = 428 (56.2%)	*Unvaccinated and* *not getting vaccinated* n = 334 (43.8%)
**Parent Vaccination Status**	*Vaccinated* n = 511 (67.1%)	Group 1: Vv n = 388 (51.0%)	Group 2: Vu n = 123 (16.1%)
	*Unvaccinated* n = 251 (32.9%)	Group 3: Uv n = 40 (5.2%)	Group 4: Uu n = 211 (27.7%)

* Abbreviation: Vv, both parent and child vaccinated. Vu, parent vaccinated and child unvaccinated. Uv, parent unvaccinated and child vaccinated. Uu, both parent and child unvaccinated. Groups 1 and 4 are concordant; groups 2 and 3 are discordant. All percentages were calculated from the total sample size of 762 parents of children aged 5–17.

**Table 2:** Associations of Parent–Child Concordance with Child Age, Parent Age, and Parent Education, by Parent Vaccination Status (n = 762) *.

**(a)** Child Age						
***Age of child (years old)***	***5–8*** n = 213	***9–11*** n = 131	***12–15*** n = 249	***16–17*** n = 169	*p*-value **^†^**	
**Vaccinated parents**						
Group 1 (Vv), n = 388	20.6%	16.2%	37.4%	25.8%	<0.001	
Group 2 (Vu), n = 123	35.8%	21.1%	25.2%	17.9%		
**Unvaccinated parents**						
Group 3 (Uv), n = 40	32.5%	7.5%	37.5%	22.5%	0.13	
Group 4 (Uu), n = 211	36.0%	18.5%	27.5%	18.0%		
**(b)** Parent Age						
***Age of parent (years old)***	***18–29*** n = 52	***30–39*** n = 238	***40–49*** n = 339	***50–64*** n = 120	***65 and above*** n = 13	*p*-value ^‡^
**Vaccinated parents**						
Group 1 (Vv), n = 388	4.9% ^a^	24.7%	49.2%	19.6%	1.5% ^§^	0.001
Group 2 (Vu), n = 123	12.2% ^a^	30.9%	43.1%	12.2%	1.6% ^§^	
**Unvaccinated parents**						
Group 3 (Uv), n = 40	7.5%	37.5%	42.5%	10.0% ^b^	2.5% ^§^	0.403
Group 4 (Uu), n = 211	7.1%	42.2%	37.0%	11.8% ^b^	1.9% ^§^	
**(c)** Parent Education						
***Parent education level***	***Some*** ***high school*** n = 28	***High school*** ***(or equivalent)*** n = 152	***Some college or trade school*** n = 168	***2- or 4-year*** ***college degree*** n = 255	***Graduate*** ***degree*** n = 159	*p*-value ^††^
**Vaccinated parents**						
Group 1 (Vv), n = 388	1.5% ^c^	14.4%	17.0%	41.2%	25.8%	0.029
Group 2 (Vu), n = 123	5.7% ^c^	14.6%	23.6%	33.3%	22.8%	
**Unvaccinated parents**						
Group 3 (Uv), n = 40	7.5%	22.5%	22.5%	30.0%	17.5%	0.084
Group 4 (Uu), n = 211	5.7%	32.7%	30.3%	19.9%	11.4%	

* Percentages represent the relative frequency of each range within each of the four groups. Each row adds to 100%, with minor variations due to rounding. Abbreviation: Vv, both parent and child vaccinated. Vu, parent vaccinated and child unvaccinated. Uv, parent unvaccinated and child vaccinated. Uu, both parent and child unvaccinated. ^†^ A significant *p*-value in the subsection indicates that parents were less likely to vaccinate their younger child. ^‡^ A significant *p*-value in the subsection indicates that younger parents were less likely to vaccinate their child. ^††^ A significant *p*-value in the subsection indicates that parents of lower levels of education were less likely to vaccinate their child. ^a–c^ Cells with the same superscripted letter are not statistically different from one another when comparing their proportion in Groups 1 vs. 2 or Groups 3 vs. 4 within the vertical category, estimated by chi-square tests (*p* > 0.05). Other pairs of cells are significantly different from one another. ^§^ The subgroup size was too small to statistically compare the frequency to the opposing subgroup in a chi-square test.

**Table 3:** Proportions of Concordance Status Associated with Perceirtions of Concordance Status Associated with Perceived Social Norms *.

*Child’s Friends Getting/Got Vaccinated?*					
	*Yes* (n = 229)	*No* (n = 176)	*About half* (n = 115)	*Not sure* (n = 242)	*p*-Value ^†^
**Vaccinated parents**					
Group 1 (Vv), n = 388	44.1%	8.8% ^a^	17.8%	29.4%	<0.001
Group 2 (Vu), n = 123	22.0%	37.4% ^a^	12.2%	28.5%	
**Unvaccinated parents**					
Group 3 (Uv), n = 40	42.5% ^b^	22.5%	7.5%	27.5%	<0.001
Group 4 (Uu), n = 211	6.6% ^b^	41.2%	13.3%	38.9%	
***Parent’s Friends Vaccinating/Vaccinated Their Child?***					
	***Yes*** **(n = 278)**	***No*** **(n = 157)**	***About half*** **(n = 127)**	***Not sure*** **(n = 200)**	***p*-Value** **^‡^**
**Vaccinated parents**					
Group 1 (Vv), n = 388	55.4%	5.2%	16.8%	22.7%	<0.001
Group 2 (Vu), n = 123	24.4%	32.5%	17.1%	26.0%	
**Unvaccinated parents**					
Group 3 (Uv), n = 40	50.0% ^c^	12.5%	7.5%	30.0%	<0.001
Group 4 (Uu), n = 211	6.2% ^c^	43.6%	18.0%	32.2%	

* Each row adds to 100%, with minor variations due to rounding. Abbreviation: Vv, both parent and child vaccinated. Vu, parent vaccinated and child unvaccinated. Uv, parent unvaccinated and child vaccinated. Uu, both parent and child unvaccinated. ^a–c^ Cells with the same superscripted letter are not statistically different from one another when comparing their proportion in Groups 1 vs. 2 or Groups 3 vs. 4 within the vertical category, estimated by chi-square tests (*p* > 0.05). Other pairs of cells are significantly different from one another. **^†^** A significant *p*-value in the subsection indicates that those who did/would vaccinate their child were more likely to believe that most of their child’s friends were or would be vaccinated. **^‡^** A significant *p*-value in the subsection indicates that those who did/would vaccinate their child were more likely to believe that most of their own friends did or would vaccinate their children.

**Table 4:** Proportions of Concordance Status Associated with Self-Reported Political Orientation *.

*Parent Political Orientation*	*Democrat/* *Leaning Democrat* n = 305	*Republican/* *Leaning Republican* n = 240	*Independent/* *Unaffiliated* n = 154	*p*-Value ^†^
**Vaccinated parents**				
Group 1 (Vv), n = 388	*55.7%*	*27.0%*	*17.2%*	<0.001
Group 2 (Vu), n = 123	*34.8%*	*42.0%*	*23.2%*	
**Unvaccinated parents**				
Group 3 (Uv), n = 40	55.6%	25.0%	19.4%	<0.001
Group 4 (Uu), n = 211	22.7%	45.9%	31.4%	

* Responses of “Something Else” or “I’m not sure” were excluded from analysis. Some rows do not add to 100% due to rounding. **^†^** A significant *p*-value indicates that those who self-reported as Democrat/Leaning Democrat were more likely to vaccinate their child.

**Table 5:** Multiple Logistic Regression on Parent–Child Vaccination Concordance (Models I and II), by parent vaccination status ^a^.

	*β*-Value	Standard Error	Z-Value	*p*-Value	Odds Ratio	95% Confidence Interval
**Vaccinated Parents**						
Child age	0.37	0.17	2.18	0.029 *	1.45	[1.04–2.04]
Parent age	0.10	0.20	0.50	0.616	1.10	[0.43–1.63]
Parent education	0.13	0.15	0.89	0.375	1.14	[0.85–1.54]
Perceived child’s friends’ vaccination status	0.56	0.27	2.10	0.036 *	1.75	[1.03–2.93]
Perceived friends’ decision to vaccinate children	0.97	0.27	3.64	<0.001 *	2.64	[1.59–4.54]
Political orientation	−0.49	0.18	−2.74	0.006 *	0.62	[0.43–0.87]
(Intercept)	(−3.89)	(1.36)	(−2.86)	(0.004 *)	(0.02)	([0.001–0.29])
**Unvaccinated Parents**						
Child age	−0.42	0.31	−1.35	0.178	0.66	[0.35–1.18]
Parent age	0.33	0.39	0.84	0.403	1.39	[0.65–3.07]
Parent education	−0.41	0.28	−1.45	0.148	0.67	[0.37–1.14]
Perceived child’s friends’ vaccination status	−0.05	0.44	−0.12	0.905	0.95	[0.41–2.34]
Perceived friends’ decision to vaccinate children	−1.48	0.47	−3.14	0.002*	0.23	[0.08–0.55]
Political orientation	0.74	0.39	1.92	0.055	2.10	[1.00–4.70]
(Intercept)	(3.99)	(2.60)	(1.54)	(0.125)	(54.21)	([0.36–110.60])

^a^ The dependent variable is concordance status, such that 0 = discordant and 1 = concordant. * Indicates significance at <0.05.

**Table 6:** Simple and Multiple Logistic Regressions on Child Vaccination Status (Model III) ^a^.

	*β*-Value	Standard Error	Z-Value	*p*-Value	Odds Ratio	95% Confidence Interval
***Simple Logistic Regressions***						
Child age	0.32	0.07	4.85	<0.001 *	1.38	[1.21–1.58]
Parent age	0.32	0.08	3.88	<0.001 *	1.38	[1.17–1.63]
Parent education	0.42	0.07	6.19	<0.001 *	1.52	[1.33–1.74]
Perceived child’s friends’ vaccination status	1.32	0.12	10.75	<0.001 *	3.77	[2.97–4.83]
Perceived friends’ decision to vaccinate children	1.67	0.13	12.41	<0.001 *	5.31	[0.02–0.07]
Political orientation	−0.61	0.09	−6.74	<0.001 *	0.54	[0.45–0.65]
***Multiple Logistic Regression***						
Child age	0.47	0.14	3.37	<0.001 *	1.59	[1.22–2.10]
Parent age	0.03	0.17	0.18	0.854	1.03	[0.74–1.42]
Parent education	0.31	0.12	2.59	0.01 *	1.37	[1.08–1.74]
Perceived child’s friends’ vaccination status	0.55	0.21	2.62	<0.001 *	1.74	[1.14–2.62]
Perceived friends’ decision to vaccinate children	1.25	0.21	5.90	<0.001 *	3.51	[2.34–5.39]
Political orientation	−0.58	0.15	−3.86	<0.001 *	0.56	[0.41–0.75]
(Intercept)	(−5.35)	(1.13)	(−4.74)	<0.001 *	0.005	([0.0005–0.04])

^a^ The dependent variable is child vaccination status (or parent decision to vaccinate their child), such that 0 = child is not vaccinated and 1 = child is vaccinated. * Indicates significance at <0.05.

**Table 7:** Multiple Logistic Regression on Child Vaccination Status (Model IV) ^a^.

	*β*-Value	Standard Error	Z-Value	*p*-Value	Odds Ratio	95% Confidence Interval
Child age	0.37	0.15	2.54	0.011 *	1.45	[1.09–1.95]
Parent age	0.01	0.18	0.05	0.959	1.01	[0.71–1.42]
Parent education	0.18	0.13	1.39	0.165	1.20	[0.93–1.55]
Perceived child’s friends’ vaccination status	0.42	0.23	1.85	0.065	1.51	[0.97–2.35]
Perceived friends’ decision to vaccinate children	1.14	0.23	4.97	<0.001 *	3.12	[2.01–4.97]
Political orientation	−0.55	0.16	−3.45	<0.001 *	0.57	[0.42–0.79]
Parent vaccination status ^b^	1.95	0.33	5.89	<0.001 *	7.00	[3.71–13.61]
(Intercept)	(−5.43)	(1.19)	(−4.56)	<0.001 *	(0.004)	([0.0004–0.04])

^a^ The dependent variable is child vaccination status (or parent decision to vaccinate their child), such that 0 = child is not vaccinated and 1 = child is vaccinated. ^b^ Parent vaccination status was included as an additional predictor variable. * Indicates significance at <0.05.

## Data Availability

The data pertaining to this paper are available from the corresponding author for one year from the date of publication upon reasonable request with a methodically sound proposal.
